# New insights into small‐cell lung cancer development and therapy

**DOI:** 10.1002/cbin.11359

**Published:** 2020-04-18

**Authors:** Yuwen Wang, Songyun Zou, Zhuyun Zhao, Po Liu, Changneng Ke, Shi Xu

**Affiliations:** ^1^ Department of Burn and Plastic Surgery, Shenzhen Longhua District Central Hospital, Affiliated Central Hospital of Shenzhen Longhua District Guangdong Medical University Shenzhen Guangdong China; ^2^ Division of Respiratory Medicine, Department of Medicine, The University of Hong Kong Queen Mary Hospital Hong Kong SAR China

**Keywords:** lung morphogenesis, small‐cell lung cancer, therapy

## Abstract

Small‐cell lung cancer (SCLC) accounts for approximately 15% of lung cancer cases; however, it is characterized by easy relapse and low survival rate, leading to one of the most intractable diseases in clinical practice. Despite decades of basic and clinical research, little progress has been made in the management of SCLC. The current standard first‐line regimens of SCLC still remain to be cisplatin or carboplatin combined with etoposide, and the adverse events of chemotherapy are by no means negligible. Besides, the immunotherapy on SCLC is still in an early stage and novel studies are urgently needed. In this review, we describe SCLC development and current therapy, aiming at providing useful advices on basic research and clinical strategy.

**Abbreviations**
ALK: anaplastic lymphoma kinase
ED: extensive‐stage disease
EGFR: epidermal growth factor receptor
LD: limited‐stage disease
NSCLC: non‐small‐cell lung cancer
SCLC: small‐cell lung cancer
TKI: tyrosine kinase inhibitors

## 1 INTRODUCTION

Lung cancer is a malignant disease and still remains a serious public health issue with high mortality and morbidity worldwide. It is estimated that approximately 2.1 million new lung cancer cases were diagnosed globally, representing 11.6% of the total cases in 2018 (Bray et al., 2018; Jemal et al., 2011). In China, lung cancer has replaced liver cancer and became the first cause of death among all the malignancies since 2008 (She, Yang, Hong, & Bai, 2013). Lung cancer is often diagnosed at an advanced stage, rendering the disease intractable. Lung cancer can be broadly categorized into non‐small‐cell lung cancer (NSCLC) and small‐cell lung cancer (SCLC) according to the histological types (Goldstraw, 2011). NSCLC represents 80% of lung cancer and can be subdivided into adenocarcinoma, large cell carcinoma, and squamous cell carcinoma, while SCLC represents approximately 15% of all lung carcinomas (Vikis, Rymaszewski, & Tichelaar, 2013). Although tobacco smoking is the primary risk factor to lung cancer, especially for NSCLC, other risk contributors including exposure to asbestos, radiation, radon gas, and environmental pollution cannot be overlooked (Chan‐Yeung et al., 2003; Choi & Mazzone, 2014; Global Burden of Disease Cancer et al., 2017).

In recent years, the emerging immunotherapies and targeted therapies open up a new realm of precision cancer treatment for lung cancer patients, especially for NSCLC (Siah, Khozin, Wong, & Lo, 2019). Currently, chemotherapy, targeted therapy, immunotherapy, radiation therapy, and surgery are the most common options of lung carcinoma therapy in clinical practice (Mott, 2018). The advancement in genetics and molecular medicine, such as epidermal growth factor receptor (EGFR), tyrosine kinase inhibitors (TKI), and anaplastic lymphoma kinase inhibitors, contributed greatly to lung cancer therapies, particularly to NSCLC (Gainor et al., 2016; Wu & Shih, 2018). Although potential targets on SCLC therapy such as poly (ADP‐ribose) polymerase (PARP), enhancer of zeste homolog 2 (EZH2), or delta‐like canonical Notch ligand 3 (DLL3) have emerged, comprehensive studies are urgently needed (Saito et al., 2018).

The morphological differences between SCLC and NSCLC could be obtained by light microscopic criteria. Generally, SCLC has a higher ratio of nuclear/cytoplasmic, finely granular nuclear chromatin, absent nucleoli, as well as common fusiform shape (Travis, 2014). SCLC always metastasizes distantly at the time of diagnosis, which abates the opportune moments to investigate evolution of tumorigenesis and gene alterations (Altan & Chiang, 2015). In that, little progress has been made in SCLC management because of the complexity of low efficient relation between the pathological characteristics and the clinical outcome. In the following sections, clinical staging, development, genetic landscape as well as current treatments in SCLC will be reviewed.

## 2 STAGING AND HISTOLOGY OF SCLC

SCLC, a special subtype of lung cancer, is characterized with good initial response to chemotherapy and radiation, aggressive proliferation, and high rate of metastasis (Dowell, 2010; Mak, Li, & Minchom, 2019; Rodriguez & Lilenbaum, 2010). According to World Health Organization (WHO) classification criterion, SCLC can be further classified into small cell carcinoma and combined subtype, in which SCLC combined with neoplastic squamous and/or glandular components. SCLC has distinct morphological characteristics including blurred borders, scant cytoplasm, finely granular “salt and pepper” chromatin, inconspicuous or deficient nucleoli, frequent nuclear molding, and a high mitotic count (Brambilla, Travis, Colby, Corrin, & Shimosato, 2001; Gibbs & Thunnissen, 2001). SCLC arising from neuroendocrine (NE) cells is one special type of NE carcinomas in lung. SCLC, associated with large‐cell NE carcinoma, intermediate‐grade atypical carcinoid, and low‐grade typical carcinoid are categorized as NE tumors (Travis, 2011). It is important to recognize the variant forms of SCLC, as patients with variant morphologies might have unfavorable prognosis. The variant SCLCs have discordant expression of the biochemical markers compared with classic SCLCs. The variants still have high concentration of brain isozyme of creatine kinase, significantly lower concentrations of neuron‐specific enolase (NSE), but lack L‐dopa decarboxylase and bombesin‐like immunoreactivity (Carney et al., 1985; Gazdar, Carney, Nau, & Minna, 1985).

## 3 ORIGIN OF SCLC

In lung development, a variety of biological players have been recognized as biomarkers, which are involved from gestation period to postnatal, including a lot of neuropeptides such as serotonin, NSE, and bombesin. NE cells are the first epithelial cells that emerge in lung organogenesis and are more enriched in fetal and neonatal lungs, which indicate its important role during pulmonary evolution and development. NE cells are derived from multipotent epithelial progenitors labeled by expression of the basic helix–loop–helix (bHLH) transcription factor inhibitor of differentiation 2 (ID2; Rock & Hogan, 2011). Many bHLH proteins have lent a hand in controlling cell differentiation in various tissues (Li, Ray, Singh, Johnston, & Leiter, 2011; Yi, Yu, Yang, Miron, & Zhang, 2017). Lineage tracing study on ID2‐positive expression cells demonstrated that ID2 could induce all the respiratory epithelial cell types (including pulmonary neuroendocrine cells [PNECs]) in the mouse model (Rawlins, Clark, Xue, & Hogan, 2009).

Pulmonary neuroendocrine cells (PNECs), occur as solitary cells in proximal airways or in clusters formed neuroepithelial bodies (NEBs) in intralobar airways. PNECs can be discovered in many species ranging from primitive amphibians to mammals, but they account for very low proportion in respiratory cell populations (Van Lommel, 2001). As the role of PNECs in lung development, many researchers concluded that PNECs exert effect via regulating amine and peptide to modulate lung growth and maturation in early stages and act as airway chemoreceptors in fetal and postnatal period (Van Lommel, 2001). PNECs have characteristic properties of both neuronal and endocrine cells. For example, neural cell adhesion molecule (NCAM1) and mammalian achaete‐scute complex homolog‐1 (MASH1), a key determinant of neuronal differentiation and maturation, were highly expressed in PNECs cells (Linnoila, 2006). Besides, NEBs are innervated by intraepithelial nerve fibers and they can sense external stimuli, such as hypoxia and nicotine, and transmit these signals to the central nervous system. At the same time, the secretory products of PNECs, including calcitonin gene‐related peptide, serotonin and bombesin, are thought to regulate epithelial cells, immune function, oxygen sensing, and effect of airway tone and blood flow (Linnoila, 2006; Van Lommel, 2001). In the latest report, Chen et al. (2019) described a new approach for the transformation of human pluripotent embryonic stem cells into NE tumors of the lung closely resembling human SCLC. They illustrated that inhibition of Notch signaling pathway could induce up to 10% lung progenitor cells to form PNECs, whose proportion could be increased by reducing the expression of retinoblastoma.

**Figure 1:**
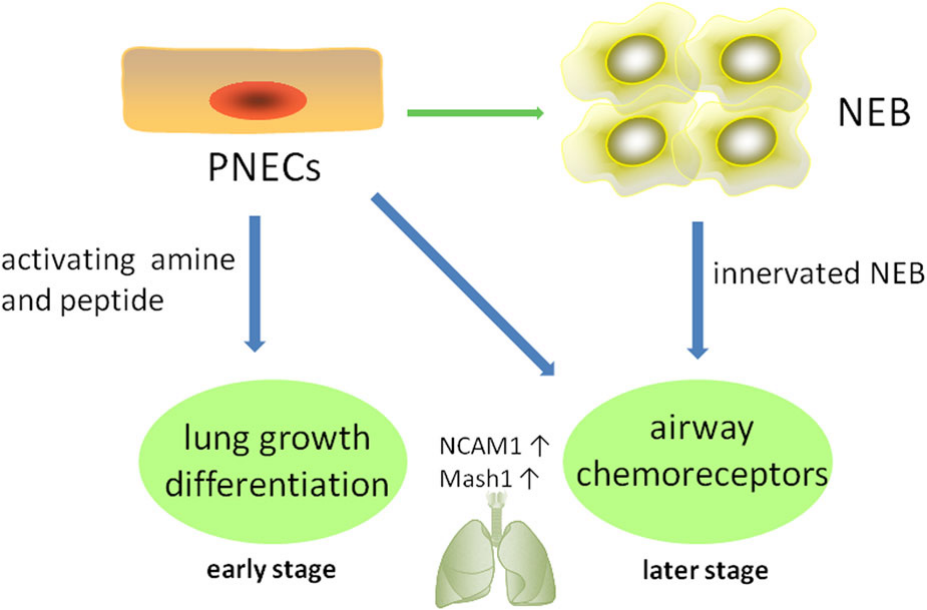
The role of PNECs in lung development. NCAM1, neural cell adhesion molecule; NEB, neuroepithelial body; PNEC, pulmonary neuroendocrine cell

Although PNECs are specified at an early stage of lung morphogenesis, which indicates their progenitor function; silencing PNECs in achaete‐scute homolog 1 (*Ascl1*, also called *Mash1*)‐deficient mice cannot stop the differentiation and maturation of other types of respiratory epithelial cells, such as secretory cells and alveolar cells (Borges et al., 1997). Indeed, a lot of studies provide evidence of their functions in both developing lungs and harmed adult lungs. It has been reported that club cells in developing lungs are associated with NEBs, which can directly contact with or secrete paracrine factors to support adjacent epithelial cells. And epithelial cells close to NEBs can be labeled with ^3^H‐thymidine, which can be used to study the progress in division. An increase in the distance from the NEBs can reduce the number of cells marked with ^3^H‐thymidine (Van Lommel, 2001). PNEC hyperplasia is also observed after pathological or external damage due to tobacco smoke, oxidant stress, nitrosamines, and burn injury. Similarly, an acute injury induced by naphthalene exposure, which can selectively decrease the amount of club cells, can increase the number of NEBs in mouse airway (Stevens, McBride, Peake, Pinkerton, & Stripp, 1997). The variant club cells, which were neighboring NEB cells, could restore the injured epithelium (Reynolds, Giangreco, Power, & Stripp, 2000). And a latest study revealed that only rare NE cells, typically 2–4 per cluster, function as stem cells that give rise to SCLC (Ouadah et al., 2019). Thus, these studies proved that PNECs or NEBs have a crucial function in the progenitor cells (Figure 1).

Researchers found that PNECs' fate specification is regulated by interaction of bHLH activator and repressor genes. The bHLH factor, such as *Mash1*, promotes NE terminal differentiation, while hairy and enhancer of split 1 (*Hes1*) inhibits this signaling pathway by suppressing the *Mash1/E2A* complex formation and repressing *Mash1* (Ito, Udaka, Okudela, Yazawa, & Kitamura, 2003). Notch signaling pathway also plays an essential role in PNEC lineage specification. Notch ligand delta‐like‐1 (*DLL1*) is observed in presumptive NE cells in proximal airways after E13.5, and its activation might be under the regulation of *Mash1* (Post, Ternet, & Hogan, 2000). Therefore, the interaction of bHLH factors and Notch signaling pathway has significant effect on pulmonary NE lineage specification.

## 4 GENETIC LANDSCAPE IN SCLC

The gene mutations identified in cancers are vital to tumor development. Comprehensive whole genome study on oncogenic driver mutations for SCLC is currently making slow progress in comparison with other kinds of cancer because of limited number of patient samples available for research. Genetically engineered mouse models for SCLC based on deletion and/or activation of known driver mutations are crucial for translational research (Gazdar et al., 2015). The most notable gene alterations discovered in patients with SCLC are almost ubiquitous loss of tumor suppressors *p53* and retinoblastoma susceptibility gene (*RB1*), as well as *MYC* amplification (Semenova, Nagel, & Berns, 2015). The functions of these genes will be discussed in the following section.

Comprehensive genomic analyses on patients with SCLC have indicated that the frequency of *p53* inactivation is approximately 75% to 90% in SCLC, which suggests its essential role in cancer development (Takahashi et al., 1989). The function of *p53* protein is to mainly get involved in genomic stability, apoptosis, and suppression of angiogenesis. The tumor suppressor *p53* is generally activated when cellular stress signals occur, such as DNA damage, hypoxia, and senescence; and induce cell cycle arrest and apoptosis as response (Carvajal & Manfredi, 2013). Not surprisingly, dysfunctional *p53* would tolerate genomic defect, which might result in high risks for driver mutations in future. *P53* in normal bronchial epithelium accompanying SCLC is detected mutated, which indicates that this gene alteration deserves an initial event in SCLC development (Wistuba et al., 2000). Besides, *TP73* is another novel mutation gene discovered through sequencing the whole genomes of 110 clinical tumor specimens of SCLC (George et al., 2015). And somatic genomic rearrangements of *TP73* exist in exons 2 and 3, resulting in a recognized oncogenic transcription factors that plays a dominant‐negative effect on wild‐type *p53* family members (George et al., 2015; Tannapfel et al., 2008). These discoveries hint the role of *p53* family members in tumor development of SCLC.

*RB1* is another tumor suppressor found inactivated in majority of SCLC, accounting for around 65% of SCLC cases (George et al., 2015). It was first discovered in retinoblastoma and was also absent or less abundant in many malignancies including prostate cancer, breast cancer, and lung cancer (Condorelli et al., 2018; George et al., 2015; Tan et al., 2014). The retinoblastoma protein belongs to pocket protein family members including RBL1 and RBL2. Compared with rare expression of other family members, *RB1* loss is a hallmark gene alteration in SCLC (Modi et al., 2000). One of the functions of *RB1* is the essential regulations on cell cycle via retarding the transition of G1 to S phase (Indovina, Pentimalli, Casini, Vocca, & Giordano, 2015). Moreover, the RB1 protein also has a vital role to regulate differentiation, as mutated *RB1* cannot inhibit cell cycle progression and is still capable of advancement on cellular differentiation (Sellers et al., 1998). In recent year, it was reported that *RB1* could directly interact with well‐known transcription factors, such as *Nanog, Oct4*, and *Sox2*, and suppress the pluripotency systems in somatic cells of patients with SCLC (Kareta et al., 2015). As a result, *RB1* depletion can lead to activation of these transcription factors and enhance the pluripotency properties, making cells much more aggressive in reprogramming and tumorigenesis (Kareta et al., 2015). Besides, researchers also found that loss of *RB1* in SCLC was greatly correlated with activation of *EZH2* (Hubaux et al., 2013). Strikingly, it has been revealed that high expression of *EZH2* in lung cancer was associated with tumor growth (Poirier et al., 2015). In general, the above evidence supports the fact that *RB1* loss is related to tumor development in SCLC.

The mutually exclusive amplification of *MYC* family member genes, including *MYC, MYCL*, and *MYCN*, occurs in around 20% of patient samples and also represents the most eminent overexpressed gene in SCLC (Peifer et al., 2012). Amplification of *MYC* gene can lead to tumor progression, chemotherapy tolerance, and poor clinical outcome, but the understanding of how these three *MYC* oncogenes affect the processes has not yet been determined (Bragelmann et al., 2017). It is known that *MYC* family proteins are transcription factors and can activate the expression of a series of genes, which contribute to cellular proliferation and cell cycle progression (Li et al., 2017). As paralogs, *MYC* family members share highly conserved and essential regions with structural homology, but exert different functions. For instance, CRISPR‐mediated depletion of *MYCL* or *MYCN* in mouse tumor‐derived SCLC could reduce tumor formation capacity, but *MYC* could not (Kim et al., 2016). *MYC*‐amplified cells were sensitive to Aurora kinase inhibitor in SCLC models; however *MYCL* and *MYCN* showed very slight response (Bragelmann et al., 2017; Mollaoglu et al., 2017). Interestingly, although *MYC* family members have a key role in proliferation and differentiation, overexpression of these three genes was also found to trigger apoptosis in IL3‐depleted myeloid cells (Nesbit, Grove, Yin, & Prochownik, 1998). All in all, the *p53, RB1*, and *MYC* gene alterations can potentially provide a wide range of alternatives for SCLC treatment (Table 1).

**Table 1:** Genomic alterations in small‐cell lung cancer

Gene/signal name	Mutation frequency (%)	Alteration
TP53	75–90	Loss
RB1	65	Loss
TP73	–	Loss
NOTCH	–	Downregualtion
MLL2	17	Downregualtion
MYC	20–30	Upregulation
PI3K	–	Upregulation
BCL2	75–90	Upregulation
RICTOR	10	Upregulation

## 5 MANAGEMENT OF SCLC

SCLC is an aggressive NE carcinoma with rapid tumor growth, high metastasis, and dismal clinical outcomes (Travis, Brambilla, & Riely, 2013; Wang, Zimmermann, Parikh, Mansfield, & Adjei, 2019). Although SCLC is highly sensitive to chemotherapy and ionizing radiation, the vast majority of patients may experience recurrence and the average survival time is only about 10 months (Kalemkerian et al., 2013). Moreover, very little therapeutic clinical improvement has been achieved during the past 30 years, leading to SCLC being labeled as recalcitrant cancer (Gazdar, Bunn, & Minna, 2017).

According to the proposal from Veterans Administration Lung Study Group (VALSG), SCLC staging can be categorized into two clinical subgroups: limited‐stage disease (LD) and extensive‐stage disease (ED; Murray et al., 1993). LD SCLC is referred to nodes and tumor confined within one hemithorax and can be treated by single radiotherapy portal, while ED SCLC is defined as tumor cells beyond these regions (Bradley et al., 2004). However, a majority of patients (approximately 70%) with SCLC are diagnosed at extensive‐stage; at this stage, cancer cells are always disseminated, which makes it difficult to study the evolution of tumorigenesis (Jackman & Johnson, 2005). However, controversies still focus on the criteria for LD and ED categories. VALSG believed that the tumor and nodal involvement in LD patients should be confined to one hemithorax, whereas, the International Association for the Study of Lung Cancer (IASLC) recommended that LD should include all patients without distant metastasis as well. As the treatment strategies for LD and ED patients could be different, the clinical outcome might be influenced by the stage determined. As a consequence, IASLC subsequently introduced tumor, node, and metastasis (TNM) in lung cancer staging to replace the VALSG system (Micke et al., 2002). Although TNM system is more accurate in tumor assessment, VALSG system is still widely used clinically for practical purposes. According to National Cancer Institute website, the standard treatment strategies for patients with SCLC is shown in Table 2.

**Table 2:** Standard treatment options for patients with SCLC

Stage	Standard treatment options
LD	Chemotherapy and radiation therapy
	Combination chemotherapy alone
	Surgery followed by chemotherapy or chemoradiation therapy
	Prophylactic cranial irradiation
ED	Combination chemotherapy
	Thoracic radiation/radiation therapy
	Prophylactic cranial irradiation
Recurrent disease	Chemotherapy
	Immune checkpoint modulation
	Palliative therapy

### 5.1 Limited‐stage disease SCLC

The therapeutic options in SCLC are dependent on the disease stage chosen. Approximately 30% of patients with SCLC are mostly diagnosed at LD. LD SCLC was believed as a curable disease because current treatment modality improved the median overall survival (OS) significantly (Farago & Keane, 2018). Surgical resection is not the first choice of the multimodality approach as SCLC is potentially metastasized in early stage. Surgery is an option for the patients in LD stage who are carefully staged with mediastinal sample collection and diagnostic computed tomography (CT) analysis (Altan & Chiang, 2015). Very limited data can be referred on a direct comparison of a combined multimodality approach for SCLC including chemoradiotherapy after surgery and chemoradiotherapy alone. However, recent clinical guidelines have recommended surgery for early stage disease, followed by systemic chemotherapy (Fruh et al., 2013; Hoda, Klikovits, & Klepetko, 2018). Peter et al revealed that surgical resection could improve the OS compared with chemotherapy for patients with LD SCLC, and stereotactic body radiation therapy could provide more benefits compared with conventional radiation therapy (Paximadis et al., 2018). Therefore, surgery for SCLC is still a controversial issue and prospective randomized trials are warranted.

Besides, concurrent chemoradiotherapy with thorax irradiation could offer more advantages compared with chemotherapy alone for patients with LD in long‐term survival study. The death rate was decreased by 14% and the 3‐year survival rate was improved by 5.4% in combined modality treatment group compared with patients who received chemotherapy alone (Pignon et al., 1992). A report from the National Cancer Data Base (United States) also demonstrated similar findings. In this report, over 6,700 patients with LD SCLC were employed, and the 5‐year survival rate for patients who received concurrent chemotherapy and thoracic irradiation was 13.3%, while that of chemotherapy alone group was only 5.7% (Gaspar et al., 2005). One notable observation was the excessive toxicity in combined modality arm, such as cyclophosphamide doxorubicin combination plus thoracic radiation.

Although concurrent chemoradiotherapy is the standard of care in limited‐stage SCLC, the optimal radiotherapy schedule and dose remains controversial. In the open‐label, Phase 3, randomized, superiority trial, survival outcomes did not differ between twice‐ and once‐daily concurrent chemoradiotherapy in patients with limited‐stage SCLC, and toxicity was similar and lower than expected in both regimens (Faivre‐Finn et al., 2017).

To obtain better results of concurrent multimodality treatment, researchers tried to optimize the sequence of administration, dosage, and proportion of chemotherapy and radiotherapy. In a Phase III study conducted by Japan Clinical Oncology Group, 231 patients with LD SCLC were randomly divided into chemotherapy concurrent with thoracic radiation group and chemotherapy sequential with thoracic radiation group. In this study, the median survival time in sequential and concurrent arm was 19.7 and 27.2 months, respectively. Hematologic toxicity was major side effect observed in the concurrent arm (Takada et al., 2002). Researchers also compared the twice‐daily hyperfractionated irradiation with once‐daily treatment in 417 patients with LD SCLC, who received four cycles of cisplatin plus etoposide. The 2‐ and 5‐year survival rates for patients who received once‐daily radiotherapy were 41% and 16%, respectively, as opposed to 47% and 26%, respectively, for the patients who received twice‐daily regimen. Not surprisingly, the frequency of grade 3 esophagitis in twice‐daily thoracic radiotherapy was higher than that of once‐daily group (27% VS 11%; Turrisi et al., 1999). In another randomized study comparing early administration of irradiation with late treatment, patients in the early arm obtained more benefits (Murray et al., 1993).

In fact, the majority of clinical trials that assessed the activity of chemoradiotherapy in LD SCLC are a subgroup study that enrolled SCLC patients including LD and ED because of the limited number of patients. Cisplatin plus etoposide (EP) with thoracic irradiation is the gold standard treatment for patients with LD SCLC (Altan & Chiang, 2015). Very few clinical trials were focused on substituting carboplatin for cisplatin on LD SCLC populations. Based on one randomized clinical study performed on patients with LD SCLC, a subset analysis concluded that carboplatin and cisplatin had similar effects (Skarlos et al., 1994). Another randomized clinical trial proved that gemcitabine and carboplatin is as effective as etoposide in terms of OS and progression‐free survival (Lee et al., 2009).

SCLC is characterized with easy relapse, among the relapsed patients, about one‐third has brain metastases as the first site of relapse; another one‐third has both brain and systemic metastases; and the remaining one‐third has systemic metastases. It has been shown that prophylactic cranial irradiation (PCI) could reduce the incidence of brain metastasis and increase the OS in both limited‐ and extensive‐stage patients (Auperin et al., 1999; Slotman et al., 2007). However, the improvement on patients who received PCI treatment came at the price of toxicity, neurocognitive disorder, and lower quality of life. In recent years, PCI treatment has met challenges due to improvement in modern imaging technique, which has individualized and systemic therapy (Farrell et al., 2018; Sio et al., 2018). As most of PCI clinical trials were conducted before the advent of modern imaging such as computed tomography (CT) or magnetic resonance imaging (MRI), the role of PCI treatment is still controversial. For example, a randomized clinical trial conducted with or without PCI in ED SCLC patients conclude that PCI could reduce the risk of brain failure and improve survival (Slotman et al., 2007). Whereas, new findings from Takahashi et al indicated the median survival in PCI arm and non‐PCI arm was 11.6 months and 13.7 months, respectively. In this randomized trial, all patients received brain MRIs before registration (Takahashi et al., 2017). Both these studies indicated that PCI could prevent brain metastases, but it might not be helpful in improving survival.

### 5.2 Extensive stage SCLC

Compared with LD SCLC, extensive stage small‐cell lung cancer (ED SCLC) was thought as an unamendable disease. Palliative care on SCLC is expected to prolong the survival time, improve quality of life, as well as minimize the risk of symptoms associated with disease. Though some new drugs have emerged in recent years, combined chemotherapy is still the footstone in all stages of SCLC (Pelayo Alvarez, Gallego Rubio, Bonfill Cosp, & Agra Varela, 2009). The first‐line treatment in SCLC recommended by the United States and Europe is 4‐6 cycles of etoposide plus cisplatin or carboplatin. In a variety of randomized trials reported in the past 30 years, etoposide plus cisplatin was compared as an investigational aim. The overall response rate (ORR), median progression free survival (mPFS), and median overall survival (mOS) for the cisplatin/etoposide compared with chemotherapy are shown in Table 3.

**Table 3:** Performance of first‐line platinum/etoposide in select randomized trials

Study design	No. of sub. in EP/EC	ORR (%)	mPFS (m)	mOS (m)	References
EP vs. IP	110	48 vs. 44	4.6 vs. 4.1	10.2 vs. 9.3	Hanna et al. (2006)
EP vs. ACE	141	77 vs. 72	NR	7.5 vs. 8.3	Baka et al. (2008)
EC vs. carbo/pemetrexed	455	52 vs. 31	5.4 vs. 3.8	10.6 vs. 8.1	Socinski et al. (2009)
EC or EP ± bevacizumab	50	48 vs. 58	4.4 vs. 5.5	10.9 vs. 9.4	Spigel et al. (2011)
EC or EP ± ipilimumab	476	62 vs. 62	4.4 vs. 4.6	10.9 vs. 11.0	Reck et al. (2016)
EC ± palifosfamide	94	NR	NR	10.4 vs. 10.0	Jalal et al. (2017)
EP ± bevacizumab	103	55 vs. 58	5.7 vs. 6.7	8.9 vs. 9.8	Tiseo et al. (2017)

Abbreviations: EP, platinum/etoposide; EC, carboplatin/etoposide; mOS, median overall survival; mPFS, median progression free survival; ORR, overall response rate.

On the basis of the discoveries in the abovementioned studies, there are several points that need to be noticed. First of all, none of the counterparts in these studies are superior to platinum/etoposide (EP) or carboplatin/etoposide (EC) in terms of ORR. It is no wonder that EP or EC is the priority option for patients with SCLC during the past 30 years. Moreover, the response rates to EP vary from 44% to 78%, which indicates that many patients are very sensitive to EP or EC treatment, and the symptoms are likely to improve after initial systemic therapy. Besides, the mPFS and mOS remain fairly unchanged in these studies, even though the time span is nearly 30 years. In summary, platinum/etoposide is a great and reliable regimen to obtain clinical improvement in short term, but the long‐term clinical outcome still remains poor.

There are a series of alternative drugs for ED SCLCs that can be used clinically, and most of the regimens are used as a second‐line therapy. The most common drugs used for ED SCLC are summarized in Table 4. The combination of cisplatin and irinotecan is widely used in Japan. Researchers have found that the mOS in cisplatin/irinotecan (IP) and EP is 12.8 and 9.4 months, respectively. Besides, the one‐year survival rate in IP arm is also superior to EP (58.4% vs. 37.3%) (Noda et al., 2002). But it is questionable that if this phenomenon can be observed in a larger population other than Japan. Subsequently, a randomized trial that enrolled 331 SCLC patients from Australia, Canada, and America failed to confirm an obvious advancement in treatment outcomes by IP compared with EP (Hanna et al., 2006). The bifurcation is probably due to different ethnicity.

**Table 4:** Combination chemotherapy for extensive‐stage small‐cell lung cancer

Standard treatment	Etoposide + cisplatin
	Etoposide + carboplatin
Other regimens	Cisplatin + irinotecan
	Ifosfamide + cisplatin + etoposide
	Cyclophosphamide + doxorubicin + etoposide
	Cyclophosphamide + doxorubicin + etoposide + vincristine
	Cyclophosphamide + etoposide + vincristine
	Cyclophosphamide + doxorubicin + vincristine

The toxic reaction is an inevitable event in chemotherapy. To reduce the adverse effect, clinicians sometimes tried to employ different drugs or combinations to replace the standard treatment. Substituting carboplatin with cisplatin is an alternative option to reduce the nephrotoxicity. Carboplatin and cisplatin can achieve similar clinical outcomes including ORR and OS with different side‐effect profiles. Myelosuppression is the major adverse reaction induced by carboplatin, while nephrotoxicity, neurotoxicity, nausea and vomiting are commonly observed in cisplatin treatment (Okamoto et al., 2007; Skarlos et al., 1994).

It is not advisable to add more drugs into the standard treatment, as another chemotherapeutic agent might lead to more toxicity with little or no improvement in outcomes. Attempts such as combination of paclitaxel with EP regimen caused additional adverse effect without significant clinical benefits (Mavroudis et al., 2001; Niell et al., 2005), but with an exception of one clinical trial of ifosfamide with EP treatment, the mOS with and without ifosfamide was 9.0 and 7.3 months, respectively (Loehrer et al., 1995).

Though SCLC has a good initial response to first‐line treatment, most of the patients might experience relapse with the disease being refractory (Schneider, 2008). How to manage the recurrent SCLC is a big issue to improve the outcomes. Normally, single‐agent regimen is preferred for relapsed SCLCs because multiple drugs could not bring more benefits but enhanced toxicity. The chance of being responsive to the second‐line treatment is dependent on the progression‐free interval after the initial therapy. Three‐month is a critical point for subsequent therapy. It is considered as resistant or refractory, if the interval is <3 months, and the chance of responding to second‐line treatment is pretty low (≤10%). It is considered as sensitive, if the interval is more than 3 months and if there is a possibility to increase the response rate by around 25% (Hurwitz, McCoy, Scullin, & Fennell, 2009; Schneider, 2008). A systematic analysis focused on 21 studies during 1984–2001 and enrolled a total of 1,692 patients eligible for analysis. The response rates to second‐line regimen in the sensitive disease (912 patients) was 27.7%, while only 14.8% was responsive to treatment in refractory group (780 patients), and the median OS also improved in the sensitive arm (7.7 vs. 5.4 months; Owonikoko et al., 2012).

Topotecan is approved by FDA and EMA, and is widely used in second‐line treatment. Ardizzoni et al. (1997) evaluated the clinical activity of intravenous (IV) topotecan in patients with recurrent SCLC. There were totally 47 patients who served as refractory, and 45 patients formed the sensitive group. The ORR in refractory arm was only 6.4%, while it was 37.8% in the sensitive group. The median OSs in the refractory and sensitive arms were 4.7 and 6.9 months, respectively. Subsequently, a randomized trial found that the ORRs in topotecan and CAV (cyclophosphamide, doxorubicin, and vincristine) arms were 24.3% and 18.3%, respectively, and the median OS also presented in a similar manner (25.0 vs. 24.7 weeks). This finding did not show any statistical significance, and single topotecan showed at least some efficacy compared with CAV in recurrent SCLCs.

The oral topotecan is another form used in second‐line treatment besides the IV form. Researchers found that oral topotecan could improve the median survival compared with best supportive care (25.9 vs. 13.9 weeks), and retain better quality life and greater symptom (O'Brien et al., 2006). Oral and intravenous topotecan showed similar effect on sensitive patients whose relapse interval was over 3 months (Eckardt et al., 2007).

To date, topotecan has been considered as second to none in second‐line treatment, as no studies have shown that the current chemicals have the better outcomes compared with topotecan on recurrent SCLCs (von Pawel et al., 2014). Owing to slow progress made in the third‐line treatment, very limited data could be referred. And only multiple target TKI anlotinib has been approved by National Medical Products Administration as a third‐line option for SCLC based on the ALTER1202 trial.

Immunotherapy has caught great attention on cancer treatment in recent years (Lee & Baek, 2019; Regzedmaa, Zhang, Liu, & Chen, 2019). Generally, immunotherapy is of three types: checkpoint Inhibitors, chimeric antigen receptor T cell therapy, and cancer vaccines. FDA has approved several drugs used in many types of cancers, including lung cancer (mainly NSCLC), melanoma, lymphoma, renal cancer, and bladder cancer (Nagai & Muto, 2018). Unlike the robust immunogenic tumor, SCLC lagged behind in immunotherapy in past decade. In recent years, more and more studies focused on novel therapeutic strategies for SCLC, with progress being made in unraveling the biology and microenvironment of SCLC (Sabari, Lok, Laird, Poirier, & Rudin, 2017). As most of the SCLC patients have a smoking history (Pesch et al., 2012), SCLC has a high tumor mutation burden, which offers numerous potential tumor‐specific antigens, and holds a new promise to improve the clinical outcome of immunotherapy for SCLC (Hellmann et al., 2018; Tian, Zhai, Han, Zhu, & Yu, 2019). It has been shown that immune checkpoints, such as programmed death 1 (PD‐1) or its ligand (PD‐L1) and cytotoxic T‐lymphocyte‐associated protein 4 (CTLA‐4), could expand the application on certain advanced stage of tumors including SCLC (Pakkala & Owonikoko, 2018; Tian et al., 2019). As an anti‐CTLA‐4 antibody, ipilimumab was the first immune checkpoint agent applied in SCLC. In a 3‐arm Phase 2 clinical trial on ED SCLC, carboplatin/paclitaxel, phased ipilimumab plus carboplatin/paclitaxel, and concurrent ipilimumab plus carboplatin/paclitaxel were compared. The immune‐related PFS (irPFS) in phased arm was 6.4 months, significantly prolonged compared with control (5.3 months) and concurrent arm (5.7 months). It seems that the median OS in phased ipilimumab group favored more (9.1 months control vs. 9.9 months concurrent vs. 12.9 months phased) although not statistically significant. However, enhanced toxicity and grade 3/4 toxicities (fatigue, arthralgia, and liver dysfunction) were commonly observed in phased ipilimumab treatment (Reck et al., 2013). Nivolumab, a PD‐1 inhibitor, has been earlier studied with encouraging results in combination with ipilimumab. A total of 216 patients were enrolled and randomized into four groups: nivolumab (3 mg/kg) arm, nivolumab (1 mg/kg) plus ipilimumab (1 mg/kg) arm, nivolumab (1 mg/kg) plus ipilimumab (3 mg/kg) arm, nivolumab (3 mg/kg) plus ipilimumab (1 mg/kg) arm, followed by nivolumab 3 mg/kg every 2 weeks until disease progression. The PFS in these three groups was 1.4, 2.6, and 1.4 months, respectively. And OS was 4.4, 7.7, and 6.0 months, respectively (Antonia et al., 2016; Pakkala & Owonikoko, 2018).

In the latest report, the FDA has approved the PD‐L1 inhibitor atezolizumab in combination with carboplatin and etoposide as a first‐line therapy for SCLC based on the Phase III IMpower133 trial. The mOS was improved from 10.3 months in the placebo group to 13.9 months in atezolizumab arm, and the mPFS was significantly prolonged from 4.3 to 5.2 months as well (Horn et al., 2018). Besides, the subgroup analysis of Japanese patients in Phase III IMpower133 trial addition of atezolizumab to carboplatin and etoposide was effective and well tolerated (Nishio et al., 2019). Another randomized Phase III trial (CASPIAN) explored a similar approach, which compared durvalumab (anti‐PD‐L1antibody) plus chemotherapy (platinum‐etoposide) and chemotherapy alone. The patients in combination arm benefited a lot, since the mOS in combination arm was 13 months versus 10.3 months in chemotherapy alone (hazard ratio, 0.73; 95% confidence interval, 0.591–0.909, *p* = .0047) (Paz‐Ares et al., 2019).

Although the preliminary trials on PD‐1, PD‐L1, and CTLA‐4 immunotherapy displayed encouraging outcomes with SCLC patients, it seems that the clinical efficacy of immunotherapy for SCLC was far less pronounced than that for solid tumors, such as NSCLC and melanoma (Nagai & Muto, 2018). The underlying mechanisms involved might be a low expression of PD‐L1, the downregulation of major histocompability complex molecules, and immunosuppression of regulatory chemokines in SCLC (He et al., 2017; Masuno et al., 1986; Tian et al., 2019; Zhu, Bagstaff, & Woll, 2006). Nevertheless, immunotherapy still brings about encouraging clinical benefits in SCLC treatment, but undergoing definitive studies are still needed.

### 5.3 Mechanisms of drug resistance

Drug resistance, regarded as one of the biggest obstacles in cancer therapeutics, is defined as cancer cells acquired resistance to one drug leading to resistance to other agents, which might have different structures or mechanisms (Nikolaou, Pavlopoulou, Georgakilas, & Kyrodimos, 2018). A majority of SCLC patients experience relapse, which indicates that drug resistance is a central problem for treatment of SCLC. Generally, several potential drug resistance mechanisms or biomarkers have been demonstrated including ATP‐binding cassette (ABC) transporters, suppression of apoptosis, cancer stem cell (CSC), and DNA damage and repair (Malone, Lardelli, Li, & David, 2019; Robey et al., 2018; Sen, Gay, & Byers, 2018; Xu, Lam, Cheng, & Ho, 2019).

A high expression of ABC transporters could increase the drug efflux and decrease the intracellular drug concentrations, which account for drug resistance. Human EGFR2 is upregulated in chemoresistant cell lines and considered as a therapeutic target for overcoming ABC transporter‐mediated resistance in SCLC (Minami et al., 2012). Similarly, the PARP, an important enzyme accounting for DNA damage and repair, is overexpressed in SCLC. PARP inhibitors were widely used to reduce drug resistance and improve therapy efficacy (Allison Stewart et al., 2017). Schlafen family member 11 (SLFN11) plays an essential role in the DNA damage response, and lack of expression of *SLFN11* has been linked to the resistance of cancer cells to DNA‐damaging agents (Malone et al., 2019). Inhibition of EZH2 (Enhancer of zeste homology 2) could have restore SLFN11 expression and resensitize SCLC derived of patient‐derived xenografts to DNA damage (Gardner et al., 2017).

Cell signaling pathway was also involved in drug‐resistant mechanism in SCLC. For example, the activation of WNT signaling in chemosensitive human SCLC cell lines through adenomatous polyposis coli knockdown induces chemoresistance, and chemoresistant cell lines demonstrate increased WNT activity (Wagner et al., 2018). MCAM (melanoma‐specific cell‐adhesion molecule) has been proved as a novel therapeutic target to overcome chemoresistance through the PI3K/AKT/SOX2 signaling pathway in SCLC (Tripathi et al., 2017).

CSCs are recognized as a subtype of cancer cells with differentiation potential and self‐renewal properties. They are considered as the origin of cancer cells and account for cancer recurrence after therapy and multidrug resistance (Ryoo, Choi, Ku, & Kwak, 2018). Increasing evidence illustrated that the drug resistance in SCLC was mainly attributed to the presence of CSC. CD133 and CD44 are specific biomarkers in lung CSCs. The lung cancer cells with high level of CD133 were quite resistant to chemotherapeutics drugs, and the expression of CD133 was elevated after treatment (Sarvi et al., 2014).

It is noteworthy that intratumoral heterogeneity, which gives rise to tumor cells presenting distinct molecular signatures with differential levels of sensitivity to treatment, was considered as one type of drug‐resistant mechanism (Dagogo‐Jack & Shaw, 2018). Notch signaling can exert both tumor suppressive and oncogenic role depending on the context in SCLC. Endogenous activation of the Notch pathway leads to a neuroendocrine (NE) to non‐NE fate switch. Although non‐NE cells are slow growing, these cells are relatively chemoresistant and provide trophic support to NE tumor cells. Therefore, combining chemotherapy and Notch inhibition might serve as a good option for selected SCLC patients (Lim et al., 2017).

## 6 CONCLUSIONS AND PROSPECTIVES

SCLC still remains a frustrating disease to treat, and a majority of patients who are diagnosed at ED eventually experience relapse despite of a good initial response to chemotherapy. Over the past decades, many progressions have been made to characterize molecular feature and development of SCLC. However, a few novel regimens were shown to significantly improve the clinical outcome for SCLC patients, which suggest an urgent need for identification of predictive biomarkers to guide personalized medicine therapy. Currently, a preliminary study of combination of immunotherapy with first‐line regimens might be a breakthrough in SCLC treatment, though many issues remain unaddressed. Drug resistance is a common occurrence in patients with SCLC, which requires therapeutic approaches that should be multidimensional, as cancer cells are featured with dynamic metabolism to reduce drug efficacy. The progressions in genomics techniques, liquid biopsies technologies, as well as single‐cell harvesting and genomics‐bioinformatics analyses might provide powerful tools for drug resistance investigation. Yet, an in‐depth understanding of the characteristics of SCLC including tumor immunity, immune microenvironment, intratumoral heterogeneity, and genomic profiles and development may provide scientific grounds and improve the clinical outcome of SCLC.

## CONFLICT OF INTERESTS

The authors declare that there are no conflict of interests.

## AUTHOR CONTRIBUTIONS

Substantial contributions to the conception or design of the work; or the acquisition, analysis, or interpretation of data for the work: Y. W., S. Z., C. K., and S. X. Drafting the work or revising it critically for important intellectual content: Y. W., S. Z., C. K., and S. X. Final approval of the version to be published: S. X. and C. K. Agreement to be accountable for all aspects of the work in ensuring that questions related to the accuracy or integrity of any part of the work are appropriately investigated and resolved: S. X. and C. K.

## Data Availability

The datasets used during the present study are available from the corresponding author upon reasonable request.
